# A Complementary Approach for Securing and Anti-Counterfeiting of Valuable Documents Based on Encryption of Computer-Generated Hologram

**DOI:** 10.3390/s25082410

**Published:** 2025-04-10

**Authors:** Zakaria E. Ahmed, Rania M. Abdelazeem, Yasser A. Attia, Tawfik A. Khattab, Claas Falldorf, Ralf B. Bergmann, Mostafa Agour

**Affiliations:** 1 Laser Applications in Metrology, Photochemistry, and Agriculture Department, National Institute of Laser Enhanced Science, Cairo University, Giza 12613, Egypt; zakaria.fm@jp.gov.eg (Z.E.A.); yasserniles@niles.edu.eg (Y.A.A.); 2 Central Administration for Counterfeiting and Forgery Research, Forensic Medicine Authority, Ministry of Justice, Cairo 11797, Egypt; 3 Engineering Applications of Lasers Department, National Institute of Laser Enhanced Science, Cairo University, Giza 12613, Egypt; rabdelazeem@niles.cu.edu.eg; 4 Dyeing, Printing and Auxiliaries Department, National Research Centre, Cairo 12622, Egypt; ta.khattab@nrc.sci.eg; 5 BIAS—Bremer Institut für Angewandte Strahltechnik, 28359 Bremen, Germany; falldorf@bias.de (C.F.); bergmann@bias.de (R.B.B.); 6 MAPEX Center for Materials and Processes, and Faculty 01: Physics and Electrical Engineering, University of Bremen, 28359 Bremen, Germany; 7 Physics Department, Faculty of Science, Aswan University, Aswan 81528, Egypt

**Keywords:** computer-generated holograms, document security, anti-counterfeiting

## Abstract

We present a novel approach for securing valuable documents using a complementary approach based on the encryption of computer-generated holograms (CGHs). The proposed approach utilizes the well-known iterative Fourier transform algorithm (IFTA) to generate a phase-only CGH for valuable digital and/or physical documents. The generated CGH is then secured by binary phase randomization, which is implemented using the symmetric encryption technique, exclusive OR (XOR). The reconstruction process for the calculated secured CGHs varied slightly depending on whether the documents were digital or physical. For digital documents, reconstruction was performed using a symmetric decryption key followed by an inverse Fourier transform (IFFT). On the other hand, the reconstruction of the physical document involved two additional processes: printing and scanning. To evaluate the quality of the digital reconstruction, the speckle signal-to-noise ratio (SSNR) was estimated for both printed grayscale and binary CGHs. The security analysis of the XOR-encrypted CGH was quantitatively evaluated to ensure the level of protection against various cryptographic attacks such as plaintext and brute-force attacks. The results revealed that the combination of phase CGHs and the XOR encryption/decryption provides robust cryptographic protection for valuable documents, benefiting document security and anti-counterfeiting.

## 1. Introduction

Valuable documents have security features that make them more difficult to copy, counterfeit, or alter. These documents are issued by government agencies, law enforcement, regulatory authorities, corporations, or financial institutions. The most common secured documents with security features are identity and travel documents such as driving licenses, national identity cards, passports, debit or credit cards, travel visas, and banknotes. Such documents are required in different applications to verify a person’s identity and credentials. However, the progress in image processing and printing technologies has made it easier to counterfeit and falsify documents. As a result, the identity documents would become illegitimate because anyone could forge their identity. These fake IDs are widely used and openly available on the global underground market, both online and physically. Criminals, including fraudsters and terrorists, can use them to enter an area covertly and carry out illegal activities without raising any red flags [1,2]. Therefore, the use of security techniques such as security threads, watermarks, cryptography, steganography, and electronic signatures is highly recommended for these valuable documents [3,4,5,6]. As part of the development of security procedures for valuable documents, digital security features including barcodes [7] have been introduced to combat counterfeiting. Barcodes can be represented in different forms, such as PDF417, DataMatrix, MaxiCode, Aztec, quick response (QR) code, etc. [8]. QR code [9] is a data representation technique that can be decoded by an optical scanner and a mobile application. However, the storage environment for printed paper with the QR code is not the same as the factory setting. Consequently, the code can become dirty, corrupt, or damaged, making the scanning process unreliable. These drawbacks show that the technology is not robust. Alternatively, holography is a widely used technology in document security based on the recording of the amplitude and phase of an object [10] in the form of a hologram. The creation of these holograms has been reported by Yaroslavskii and Merzlyakov [11], Dallas [12], and Lee [13]. The most widely used hologram for document security is an optically variable device (OVD). OVD is an iridescent or non-iridescent security feature that provides different information, such as color change or movement, that is affected by illumination and/or observation angle [14]. Concerning the state of the art, iridescent OVDs can be classified into two main categories: diffractive optically variable image devices (DOVIDs) and interference security image structures (ISISs) [15]. DOVIDs contain micro- or nano-structures in the form of diffractive gratings that produce optically variable effects such as dynamic chromatic, holographic, kinematic, and 2D or 3D images that are easy to recognize [16]. DOVIDs can also contain elements that are invisible to the naked human eye, such as micro printing, kinetic effects, micro text, or a variable laser-readable micro-image that is invisible under white light when magnified. The micro-image is known as the hidden image and is generated by incorporating a computer-generated hologram (CGH) into the DOVIDs [15]. Read-out of the additional CGH is done optically using coherent illumination. On the other hand, ISISs are based on thin-film structures, which leads to a color change upon tilting the document [17,18]. Thus, DOVID consists of adjacent fringes, whereas ISIS consists of one or more thin films. CGH, or digitally generated holographic interference patterns, is a technique that uses numerical approaches to mimic the actual processes behind the optical recording and reconstruction of a real hologram. Therefore, it represents a major improvement over classical holography [19]. According to the review in Ref. [20], this technique has been used to secure valuable documents in applications such as security printing and document authentication. Using a sophisticated amplitude that is undetectable to the human eye but readable under coherent light illumination, CGHs can encode images and digital data. Two types of holograms have been proposed for document security: photopolymer security holograms with integrated micro-CGHs [21] and printed holograms applied using thermal printing, laser ablation, and office printers [20]. For authentication, the micro-CGHs can include individualized encoded data. It is often possible to decode the printed CGH data using smartphone cameras based on numerical reconstruction techniques. The key advantages of CGHs for document security are their high level of security and their adaptability in encoding different types of data.

Previous studies [19,20,22,23] have used printed binary amplitude computer-generated holograms (CGHs). In holography, however, the phase distribution carries most of the information. Encoding the phase using binary amplitude is challenging because it introduces ambiguity, requiring a higher space–bandwidth product (SBP) to accurately represent the phase. This is particularly problematic in applications such as secure document authentication, where the SBP is inherently limited, restricting the performance of binary amplitude CGHs in faithfully encoding the information to be encrypted.

Image-encryption techniques have been proposed in the literature based on various methods. The utilization of chaotic systems in cryptography attracted the attention of a large number of researchers due to their sensitivity to initial conditions (butterfly effect), non-linearity, the fact that the initial parameters act as the encryption key, and unpredictability [24,25,26]. However, they are too complex to use because of their computational complexity, intensive memory requirements, and high sensitivity to key values, which can lead to decryption failure. Alternatively, the exclusive OR (XOR) encryption/decryption technique might be employed due to its simplicity and speed.

Therefore, in the current study, we aim to explore a novel approach for securing valuable digital and/or physical documents such as a driver’s license and the bio-data page of a passport. The proposed approach uses a binary phase-only CGH to overcome the drawbacks of using an amplitude hologram. In addition, the generated CGH is modified by modulating it with a random phase mask using the well-known XOR encryption/decryption technique to add a layer of protection. Thus, the proposed approach is achieved via hologram generation, XOR encryption/decryption processes, and reconstruction. The integration of hologram generation with XOR encryption/decryption provides robust cryptographic protection for valuable documents, a method that, to our knowledge, has not been used before. Unlike the purely cryptographic layer of chaotic methods, the integration of XOR with the CGH provides a hybrid security approach that combines cryptographic encryption with holographic phase encoding. This complementary approach ensures compatibility with standard printers/scanners without the need for specialized hardware. The proposed method and its evaluation are presented and discussed in the following sections.

## 2. Phase-Only Hologram Generation

**Figure 1:**
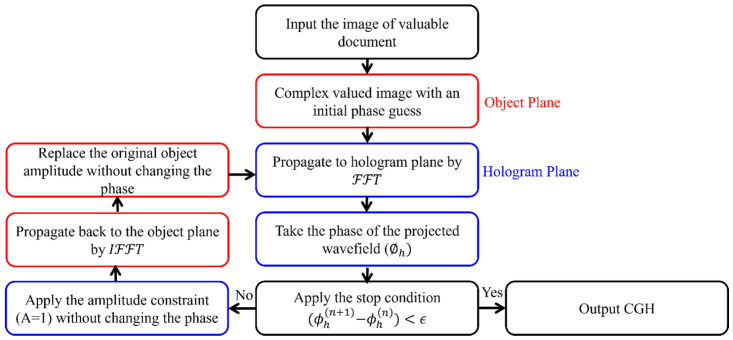
Schematic flowchart for generating a phase-only CGH using IFTA. Note that the red boxes correspond to operations performed at the object plane, while the blue boxes correspond to operations performed at the hologram plane.

CGHs encode arbitrary wavefields, amplitude, and phase information, using a computer to mathematically model the interference patterns obtained by superimposing the wavefield diffracted by an object and a known reference wave at the hologram plane. Based on numerical propagation between the object and hologram planes, the phase information computed at the hologram plane can be converted to a wavefield with an intensity that approximates the desired object when reconstructed. This allows encrypted document images, the intensity of the wavefield at the object plane, to be securely transmitted and retrieved using holographic techniques. Accurate numerical reconstruction algorithms are essential to authentically represent the details of the original input image. The flowchart in Figure 1 demonstrates the procedure used for calculating a phase-only CGH using the well-known IFTA (which refers to the Gerchberg–Saxton (G-S) algorithm) [27,28].

It should be noted that we chose the well-known G-S algorithm for CGH generation because it is a reliable and widely recognized method for encoding valuable information in holographic data. Despite being an established approach, the G-S algorithm remains highly effective due to its iterative nature and ease of implementation. Furthermore, its widespread use in similar contexts ensures that our results are reproducible and consistent with existing research in diffractive optics and holographic projection applications.

The IFTA approach uses the fast Fourier transform (FFT) and its inversion (IFFT) for forward and backward propagation between two planes, using the predefined intensities across these planes as constraints [11,29,30]. This inverse problem is then solved iteratively as follows, starting with a complex-valued object $\left(U_{0}^{\left(0\right)}\right)$, with an initial phase estimate $\phi_{0}^{\left(0\right)}=0$ and an amplitude $A_{0}=\sqrt{I}$, where *I* is its intensity constraint applied at the object plane, i.e., the information to encode as an image, which can be expressed as follows:

(1) $$U_{0}^{\left(0\right)}=A_{0}\cdot \exp\left[i{\phi_{0}}^{\left(0\right)}\right]\;.$$

The diffracted field at the hologram plane $\left(U_{h}^{\left(n\right)}\right)$ is estimated by propagating the object field, $\left(U_{0}^{\left(0\right)}\right)$ given by Equation (1), using the FFT expressed by the operator (F) in the following:

(2) $$U_{h}^{\left(n\right)}=F\left\{U_{0}^{\left(n\right)}\right\}\;.$$

At this plane, the second amplitude constraint is applied (i.e., assuming a plane wave illumination $A_{h}=1$) while maintaining the same phase ($\phi_{h}^{\left(n\right)}$), which is estimated by dividing the complex function $U_{h}^{\left(n\right)}$ by its amplitude $|U_{h}^{\left(n\right)}|$. The wavefield at the hologram plane can be written by modifying Equation (2) as follows:

(3) $${U_{h}^{\prime }}^{\left(n\right)}=A_{h}\cdot \exp\left[i\phi_{h}^{\left(n\right)}\right]\;.$$

Then, the back propagation to the object plane is performed by applying the IFFT expressed by the operator ($F^{-1}$) to Equation (3), resulting in the following:

(4) $${U_{0}}^{\left(n+1\right)}=F^{-1}\left\{{U_{h}^{\prime }}^{\left(n\right)}\right\}\;.$$

Thus, Equation (4) gives the wavefield at the object plane for the next iteration, i.e., n+1. Here, iteration n+1 is started by reapplying the amplitude constraint (i.e., replacing the original object amplitude $A_{0}$) while keeping the same phase $\left(\phi_{0}^{\left(n+1\right)}\right)$. The generated wavefield can be expressed in Equation (5) as follows:

(5) $${U_{0}^{\prime }}^{\left(n+1\right)}=A_{0}\cdot \exp\left[i\phi_{0}^{\left(n+1\right)}\right]\;.$$

The iteration procedure is repeated until no change in phase is observed or a predefined condition $\left(\phi_{h}^{\left(n+1\right)}-\phi_{h}^{\left(n\right)}\leq \epsilon \right)$ is satisfied.

In the current study, the CGHs were generated from grayscale images. This can be attributed to three reasons: (i) practical compatibility, (ii) computational efficiency, and (iii) standardization. (i) Practical compatibility: most laser printers and scanners are commonly optimized for monochrome/grayscale output, ensuring reliable binarization and reconstruction. (ii) Computational efficiency: grayscale CGHs reduce computational complexity by 3× compared to RGB-based holograms, which require separate phase calculations for each color channel. (iii) Standardization: security features like MRZ text and signatures are typically monochrome in official documents, aligning with grayscale encoding. It should be noted that several propagation operators based on Fresnel and plane wave compensation can also be used [31,32]. In addition, the hologram itself could be modulated within the hologram generation step, employing spatial multiplexing, analogous to [33], to encode multiple pages of information in a single CGH. In the current study, the CGH generation and reconstruction processes were implemented using MATLAB R2021a for numerical simulations and PyCharm Professional 2023.2 for developing a user-friendly graphical interface. The generation process involves calculating the CGHs using IFTA, in addition to the XOR encryption for securing the calculated CGH. On the other hand, the reconstruction process includes the reconstruction of the calculated CGHs using inverse IFTA along with XOR decryption. It is worth mentioning that the elapsed time of generating and reconstructing the CGH is highly influenced by the specifications of the used central processing unit (CPU). In the current study, the calculation time required to generate the CGH after 100 iterations using Core^TM^ i5-3210M CPU @ 2.50 GHz with 4 GB RAM, operating system Windows 10 pro-64-bit, was 3.98 s, and the elapsed time for the XOR encryption was 0.20 s. On the other hand, the reconstruction time for CGH, including the XOR decryption step, was 1.55 s.

## 3. Results and Discussion

The proposed method for securing valuable documents covers both digital and physical formats. For both types, document security is achieved by generating a CGH and adding an extra layer of protection through randomization with a binary phase mask.

This randomization can be implemented using basic symmetric encryption techniques such as XOR. Although XOR is a basic encryption technique and may be vulnerable to attack, the use of a long randomized encryption key makes it able to deter this type of attack. To the best of our knowledge, the combination of CGH and XOR encryption/decryption offers a strong cryptographic protection method that has not been applied to valuable documents. To decrypt the secured CGH, one must first apply XOR decryption, followed by an IFFT.

The proposed approach for adding a layer of protection to binary CGH is based on integrating it with encryption and decryption using the XOR operation that is part of symmetric key encryption. This ensures that the same key used for encryption is also used for decryption, which is a simple and effective way of protecting image data. If we assume that the binary image, represented as I[m,n], is then XORed with a generated binary key K[m,n] of the same dimensions, *m* and *n*, then the encryption is mathematically described as follows:

(6) $$I_{enc}\left[m,n\right]=I\left[m,n\right]⊕K\left[m,n\right]\;.$$

In Equation (6), $I_{enc}\left[n,m\right]$ is the encrypted binary CGH and ⊕ refers to the XOR operation applied to each pixel according to the basics of the XOR operation. Decryption is then performed by applying the XOR decryption algorithm, this time between the encrypted CGH image $I_{enc}\left[m,n\right]$ and the original key K[m,n] as the decrypted key. This process is described as follows:

(7) $$I_{dec}\left[m,n\right]=I_{enc}\left[m,n\right]⊕K\left[m,n\right]\;.$$

The operation given in Equation (7) restores the optimal distribution to the original encrypted binary CGH, I(x,y). The decrypted CGH image is then stored and can be visually compared to the original image to confirm the accuracy of the process. This method is particularly valuable for binary images such as CGH due to its simplicity and ability to effectively secure data with minimal computational overhead.

### 3.1. Secured CGH of a Digital Document

For validating the proposed method, a template of a designed digital driving license was tested. A digital driving license is one of the commonly used documents to authenticate the identity of a license holder [34].

#### 3.1.1. CGH Generation of Digital Documents

**Figure 2:**
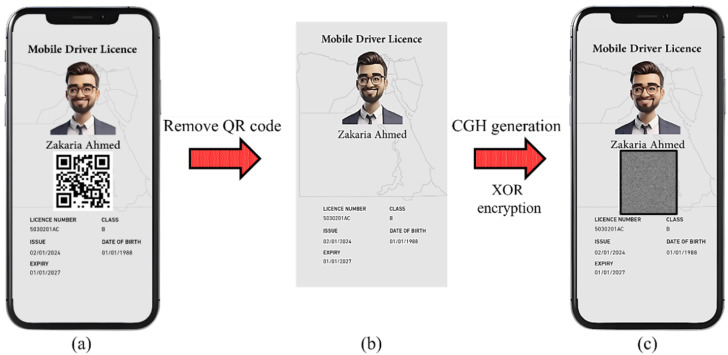
(**a**) A designed template of a digital driver’s license with a QR code and size of 1730 × 1060 pixels. (**b**) The driver’s license after removing the QR code and (**c**) replacing the QR code with the CGH of the image in (**b**). Note that the actual size of the CGH image in (**c**) is 1730 × 1060 pixels.

The simulation of replacing the commonly used QR code with a CGH in a designed digital driver’s license is shown in Figure 2. As shown in Figure 2a, the QR code is used as a security feature. However, reading the dynamic QR code requires an internet connection for authentication. Therefore, instead of the QR code, CGH can be used to secure the extracted region of interest in Figure 2b, as depicted in Figure 2c. The CGH is computed for the full data image shown in Figure 2b.

The QR code is used to store specific information from the digital document, i.e., text and/or numbers, but it cannot store an image due to its limited data storage. In contrast, CGH can store text, numbers, and the full image of a document. The authentication process of the driving license containing a dynamic QR code requires an internet connection. The advantage of using CGH instead of the QR code is its ability to store a vast bundle of data (i.e., full image storage) and the verification of identity without the need for a database connection to retrieve and compare the data as with the QR code. Additionally, the CGH-encoded data can be retrieved offline.

#### 3.1.2. XOR Encryption

In addition, an extra layer of protection was added to the binary computed CGH using one of the basic symmetric cryptographic XOR techniques [35]. It is worth noting that although the XOR method is a basic symmetric cryptographic technique, it was chosen for its practicality and efficiency in implementing an additional layer of protection for the computed CGH. This method has several advantages that make it superior for securing the CGH. It can be used for real-time document verification with minimal computational cost. In addition, the use of a long, randomly generated encryption key enhances security by making the encryption difficult to break. Combined with the CGH, XOR provides an effective layer of protection for valuable documents against unauthorized access.

Because XOR encryption works with binary data, it is particularly useful for processing digital images commonly encoded in binary format. In this method, each binary bit of the matrix of the binary CGH matrix is XORed with a corresponding bit of a binary key. By comparing each pair of bits, the XOR process creates a ciphertext that hides the original image data (encryption process). The XOR operation on the ciphertext is performed with the same unique key during the decryption process (reserving the encryption process), as demonstrated in Table 1, revealing the original CGH image. Thus, the XOR encryption/decryption process is easy to use, computationally efficient, and provides an additional protection layer to the computed CGH.

There are several advantages of using XOR encryption to secure CGH images. The confidentiality of CGH images is enhanced by XOR encryption, which provides a quick/fast and easy way to obfuscate image data. Furthermore, because XOR operations are computationally efficient, they can effectively encrypt large amounts of image data without acquiring significant processing overhead. Similarly, XOR encryption is easy to integrate and deploy in current systems or software used for CGH generation processing. Despite its simplicity, XOR encryption can offer a basic level of security for CGH images, particularly if a suitably long, strong, and randomly generated encryption key is used. It should be noted that integrating the encryption key into CGH does not change the amount of storage required, as the result of this process is also a 1-bit image with the same resolution. 

#### 3.1.3. Security Analysis of XOR-Encrypted CGH

To assess the security strength against cryptographic attacks, we used entropy analysis, measurements of horizontal correlation, the number of pixels change rate (NPCR), and unified average changing intensity (UACI) [36], as shown in Table 2.

##### Entropy Analysis

The measurement of cryptographic image security against attacks relies on entropy. The entropy is defined as how randomly distributed the pixel values are in an encrypted CGH image, where higher entropy indicates better randomization of the encoded data, making it is difficult for attackers to identify or predict the statistical pattern required to retrieve the original information. This analysis was applied to two different encrypted images (image 1 and image 2), as shown in Table 2. Both images reveal high entropy values of (1). The results obtained ensure robust protection against statistical attacks.

##### Correlation Coefficient Analysis

The relationships between pixels were assessed using the correlation coefficient. When the images lack encryption, the structural elements of pixel arrays create strong links between adjacent pixels. In contrast, during the encryption process, the relationship between the pixels is greatly reduced. As illustrated in Table 2, a low correlation of −0.0012 and 0.0010 was obtained between the original image and the encrypted two images (i.e., image 1 and image 2), which ensures difficulty for attackers trying to retrieve the original information.

##### NPCR and UACI Analysis

Evaluation of the sensitivity of the encryption process to small changes in the plaintext image depends on the NPCR and UACI metrics depicted in Table 2. The NPCR determines the proportion of pixel change in the corresponding encrypted images from nearly identical original images. UACI analyses average intensity differences. A perfect encryption system of a grayscale image achieves an NPCR threshold of 99%, meaning that each input pixel responds differently to small adjustments in the original image. For binary CGH images, an XOR encryption process produces an expected NPCR rate of 50% because pixels exist in a two-state system between 0 and 1. The NPCR for the two encrypted images (i.e., image 1 and image 2) is 50.0338%, demonstrating the correct functioning of the diffusion process. A UACI value of 50.0338% shows that minimal changes in plaintext will produce substantially different ciphertext values.

##### Security Implications Against Brute-Force Attacks

Brute-force attack is a hacking technique that involves repeatedly trying different combinations of encryption keys until the correct one can be obtained. Although our proposed algorithm was based on XOR encryption, which is easy to implement and has poor cryptographic protection, we used two mechanisms to make it more resistant to such attacks. Such mechanisms involve the addition of salt to the master key and spatial shuffling. The master security key was generated from Password-Based Key Derivation Function 2 (PBKDF2) using Secure Hash Algorithm 256-bit that performs 1000 iterations for generating 128-bit cryptographic salt. For the CGH with a size of M×N pixels, the spatial shuffling generates M×N possible shuffles that combine with the 256-bit key space and contribute $2^{256}$ potential combinations. Let us assume that the attacker has a supercomputer that can check $10^{18}$ keys per second, which is optimistic. Therefore, the attacker will need $3.7\times 10^{51}$ years to complete the decryption process. These two procedures strengthen the security against brute-force attacks, making it extremely challenging for attackers to simultaneously break both the generated salt and the spatial shuffling of the XOR-encrypted CGH.

#### 3.1.4. CGH Decryption and Reconstruction of Digital Documents

**Figure 3:**
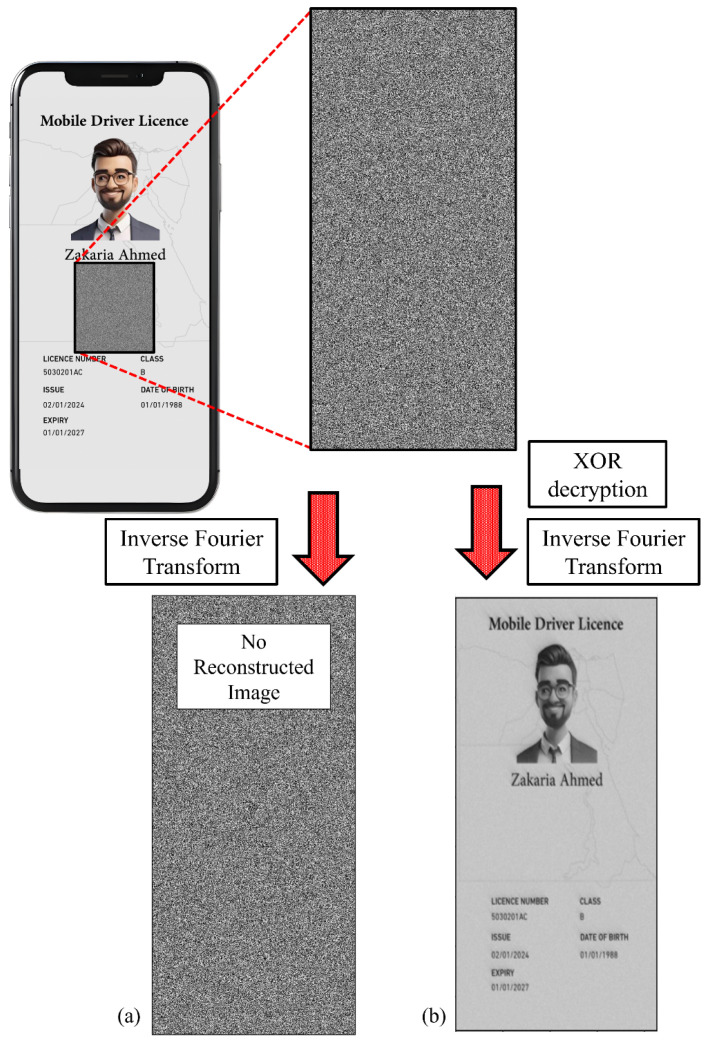
(**a**) The reconstruction process of digital driver’s license CGH without applying the decryption process and (**b**) the decryption process followed by the reconstruction process. Note that in (**a**), the reconstruction does not provide any information about the encoded license, while in (**b**), one can retrieve all encoded license information. It is noted that the size of the driver’s license and CGH images is 1730 × 1060 pixels.

Before starting the reconstruction process of the encrypted CGH image, the XOR decryption key should be applied to decrypt the encrypted CGH image. This process is called decryption. Based on Equation (7), the XOR decryption step performs XOR decryption between the secured binary CGH and the original encryption key. Hence, the original binary CGH will be retrieved. Consequently, another process was applied, which is called reconstruction. This process involves applying IFFT to the binary decrypted CGH to obtain the original image of the valuable document. Both processes, i.e., decryption and reconstruction, are demonstrated in Figure 3. The results of the reconstruction show that the encoded license information can only be fully retrieved with good quality if the correct encryption key is used to decrypt the secured CGH.

### 3.2. Secured CGH of a Physical Document

**Figure 4:**
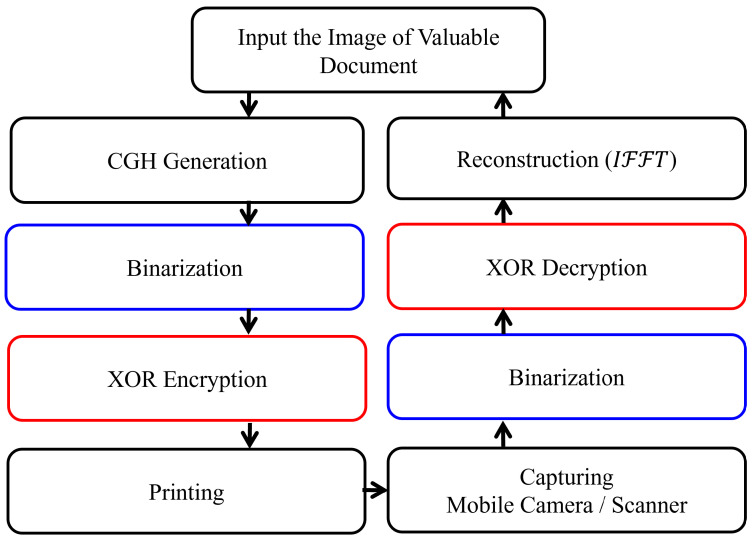
Flowchart of the procedure of securing valuable physical documents using the combination of CGH and XOR encryption/decryption.

On the other hand, the process of securing a physical document involves more steps, as shown in Figure 4.

#### 3.2.1. CGH Generation of Physical Documents

The calculated phase-only CGH using IFTA for securing a valuable document is obtained as a grayscale image with values ranging from 0 to 255. To overcome the resolution limitations of the used printers and the limited pixel size, the CGH is binarized using Otsu’s method [37], resulting in a matrix of 0 and 1 values. To increase the security of the binarized CGH, a protection step is introduced by randomizing it using the XOR encryption key. The encrypted CGH is then printed on secured or standard office paper. Once printed, the secured CGH can be captured with a smartphone camera or scanned with a scanner, then binarized. The final step in securing the valuable physical document is to numerically reconstruct the image. This process requires the application of the XOR decryption key with the original encryption key, followed by an IFFT to obtain the best approximation of the original input image. Please note that when configuring the printing process, it is essential to ensure that there is no scaling or difference in resolution between the printed and scanned images of the secured CGH.

We examined safeguarding the bio-data page information of a passport, as delineated by the standards of the International Civil Aviation Organization (ICAO). This is done by extracting specific data fields from the passport image, such as the passport number, expiration date, passport holder image, signature, or name of the passport holder. Additionally, the bio-data page of the passport and/or the entire image of the machine-readable zone (MRZ) can be captured and encrypted as CGH.

**Figure 5:**
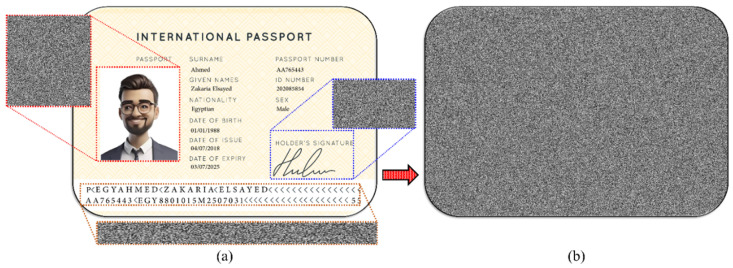
(**a**) A designed passport bio-data page with a size of 1270 × 1870 pixels and selected regions of interest to calculate their CGHs. (**b**) The CGH of the full bio-data page with a size of 1270 × 1870 pixels.

The process of generating CGHs for selected parts of the bio-data page of a designed passport template is shown in Figure 5. The chosen parts used for calculating CGHs are the personal photograph, the signature of the passport holder, and the MRZ. The CGH of each selected part of the passport (shown in Figure 5a) was calculated using the IFTA. The CGH can be generated for the entire bio-data page, as depicted in Figure 5b.

#### 3.2.2. Printing of the CGH

##### Grayscale CGH

A monochrome laser printer was used to print different sizes of generated grayscale CGHs. Grayscale CGH is an image represented in 8 bits, which means that it uses 256 ($2^{8}$) grayscale values ranging from 0 (corresponding to black color) to 255 (corresponding to white color). The potential problem with printed grayscale CGH images is the low contrast ratio. This arises from the printer’s difficulty in achieving sharp distinctions between different grayscale levels, resulting in a washed-out or unclear appearance. This also makes it sensitive to noise during scanning, such as dust and uneven lighting. The noise during the scanning process arises from dust particles that may settle on the document or scanner; they obscure small portions of the printed CGH. This results in the introduction of unintended “black spots” or “voids” in the scanned image, which distort the reconstruction process. Additionally, uneven lighting while using a scanner or a smart phone camera causes the tested document to reflect too much light; this introduces variations in pixel intensities that are unrelated to the actual hologram pattern, effectively adding random noise to the CGH. This in turn leads to poor numerical reconstruction of these CGHs. This problem is demonstrated in Section 3.3 showing the degradation of the reconstructed image.

Therefore, to overcome these drawbacks, the CGH should be converted into a binary one during the computation process.

##### Binarized CGH

Binarization is performed during the CGH calculation. This was achieved using the Otsu threshold technique [38,39]. This method is an automatic image thresholding technique in which the histogram of the grayscale CGH is calculated. This is then iterated for all threshold values from 0 to 255. For each threshold, the histogram was divided into two classes of pixels: pixels with intensities below the selected threshold value and pixels above the selected threshold. Hence, the variance between the two classes should be estimated for each threshold. The maximum variance between the two classes is chosen as the appropriate value to obtain the best separation between the two classes. Finally, based on the selected threshold value, the pixels below the threshold are set to 0 (black), and the pixels above the threshold value are set to 1 (white). A binarized phase-only CGH offers an advantage over a grayscale one due to its higher contrast ratio. This is because laser printers use a laser to selectively charge the drum, enabling toner to adhere only to the exposed areas through charge neutralization [40]. The difference between the presence and absence of toner on the surface of the drum is more pronounced than the thin variations in grayscale intensity. The higher the contrast, the sharper the image, and this affects the scanning processing of printed images when illuminated during the scanning process. The scanning process, based on light, triggers the document, then the reflected light indicates the presence or absence of toner on the surface of the paper, which is easier to detect in binary images than in grayscale images.

The binarization process reduces the storage space of the 8-bit CGH to one-eighth, which could be an advantage. However, it also reduces the bandwidth product of the gray CGH to one-eighth [41], introducing quantization errors. These errors appear as noise in the background and reduce the contrast of the reconstructed encoded information. In addition, the quality of the holographic reconstruction is affected by the reduction of the bandwidth product by one-eighth due to binarization [42]. However, we mitigated this degradation by applying a low-pass filter to suppress these artifacts and to improve the visual quality in terms of contrast and SNR of the reconstruction, similar to [43,44]. Despite these trade-offs, binarization simplifies the encryption process, making it more efficient and secure. Moreover, it enhances compatibility with the printing and imaging stages, allowing for seamless integration into document security.

On the other hand, conversion of the grayscale image to a binary image reduces the speckle noise, which is a common error that can occur during the reconstruction of the CGH, resulting in a cleaner and more consistent reconstructed image. Despite the advantages of binarization, there is a serious drawback, which is the formation of double-overlapped images during reconstruction. To overcome this problem, the CGH generation code has been modified. The modification can be made in one of two ways to overcome this obstacle. First, the modification of CGH during the generation process is a linear phase, i.e., a ramp. Second, a zero-padding image is created to become the base of the input image.

#### 3.2.3. Scanning of the Printed CGH

The printed CGHs were scanned using two different methods, i.e., a mobile phone camera and a scanner. The camera of a Xiaomi Poco X3 NFC (Xiaomi, Beijing, China) smartphone with specifications of Android 12, MIUI 14.0.2, 64 MP, f/1.9, (wide), $1/1.73^{\prime \prime }$, 0.8μm, PDAF, 13 MP, f/2.2, $119^{{}^{\circ}}$ (ultrawide), 1.0μm, 2 MP, f/2.4, (macro), 2 MP, f/2.4, (depth), dual-LED flash, HDR, and panorama was used to capture the printed CGHs. Each CGH was captured with different modes: photo AI, original document, black and white document, and enhanced document. It should be noted that the images captured by the mobile phone in these different modes had a bit depth of 24 bits and were represented as colored images (RGB). On the other hand, an optical scanner model HP ScanJet Pro 2500 f1 (HP, Beijing, China) accurately captures text from documents for easy editing with HP Scan and I.R.I.S. Readiris™ Pro OCR software (HP, China) producing sharp, true-to-life scans of documents, graphics, and photos at up to 1200 dpi resolution. It has been used to scan printed CGHs on paper. The scanning process can be in either color or monochrome modes. The scanned image produces a bit depth of 8 and 24 bits in the color mode. In the monochrome mode, the bit depth is 1 bit. Therefore, in our case, it was preferable to use the monochrome mode rather than the color mode. This is because the image captured with the monochrome mode is a 1-bit binary image, so it does not need to be re-binarized after capture but is directly subjected to reconstruction. However, images scanned using the color mode and those captured by a smartphone camera must be binarized before the numerical reconstruction to remove noise for better reconstruction.

#### 3.2.4. CGH Reconstruction of Physical Documents

**Figure 6:**
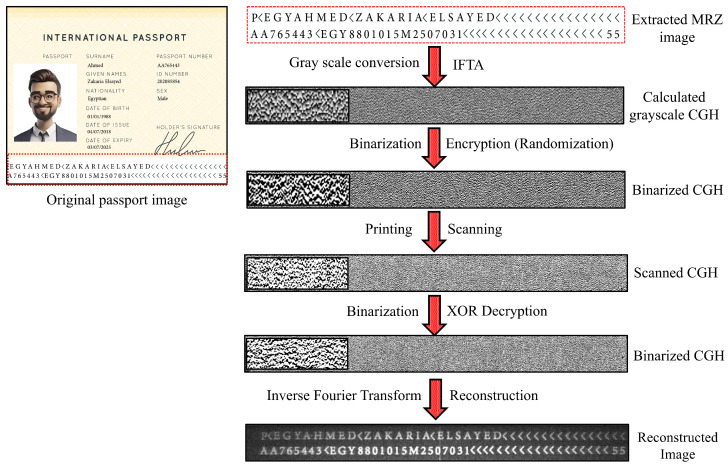
The procedure of generating and reconstructing the MRZ image of a designed passport bio-data page of a physical document. Note that the MRZ image is resized to 200 × 600 pixels before CGH generation, the actual size of the printed CGH is 1×4 inches (25 mm × 100 mm) (600 dpi), and the printed CGH is scanned by a 600 dpi scanner.

An essential step after capturing the CGH image of a physical document, either with a mobile camera or a scanner operating in color mode, is the binarization process. Binarization is the process of converting the captured or scanned images into black and white pixels, removing background variations and irregularities in lighting. This, in turn, makes it easier to distinguish between text or features of interest and background. As a result, the quality of the numerical reconstruction of the physical documents is improved. The flowchart of the CGH reconstruction of the physical documents is shown in Figure 6. It is worth mentioning that the captured 1-bit image did not need to be binarized because its pixels already had values of (0,1). In addition, the MRZ intensity reconstruction is modulated with an intensity envelope function, i.e., the center is brighter than the image sides, derived from the Fourier transform of a window function. Finally, the reconstruction is modulated with a sinc function, which is used as described in [45] because of the limited pixel size.

One of the most common document security features used worldwide is the smart chip. The smart chip is typically embedded within the cover or one of the inner pages of biometric passports. It contains the holder’s biometric data, such as fingerprints, facial recognition, and iris patterns, along with other identifying information, and it plays a crucial role in authentication during border control. Smart chips are often difficult to access, but in cases of forgery, fraudsters may attempt to tamper with or physically destroy the chip to prevent electronic verification. However, smart chips are relatively expensive to produce and integrate into documents.

Alternatively, CGH offers the storage of photos, text, and numbers like a smart chip but at a lower cost, and they can be accessed offline. Furthermore, it can be reconstructed even if part of it is cut off. Therefore, CGH can be used as an effective tool in place of a smart chip.

### 3.3. Factors Affecting the Quality of the Reconstruction

**Figure 7:**
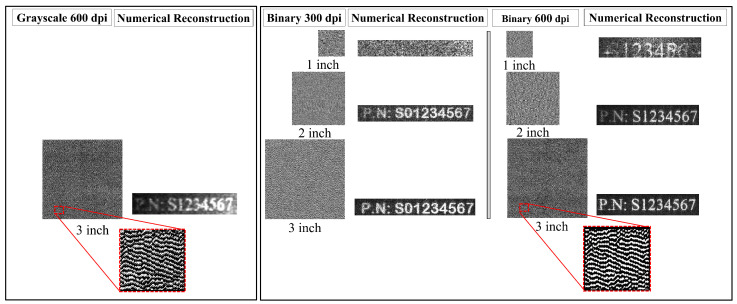
The procedure for generating and reconstructing a CGH of a designed passport number of a physical document. Note that the passport image is resized to 600 × 600 pixels before CGH generation, the actual size of the printed CGH is 2 inches (50 mm × 50 mm) (600 dpi), and the printed CGH is scanned using a 600 dpi scanner.

The quality of the digital reconstruction of a physical document was evaluated based on the printing resolution and the size of the printed CGH. First, we tested the quality of the reconstruction of printed grayscale CGH with a printing resolution of 600 dpi. As mentioned in “Section Grayscale CGH”, the reconstruction quality of the printed grayscale CGH is quite low, as shown in Figure 7. Then, we tested the quality of the printed binary CGHs.

**Figure 8:**
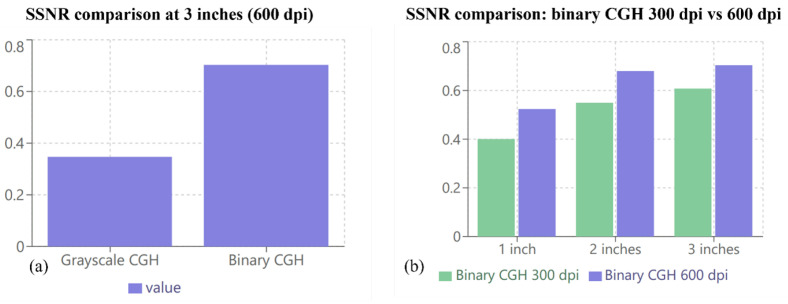
The estimated values of the speckle signal-to-noise ratio (SSNR) for (**a**) the grayscale versus binary CGHs at 3 inches (600 dpi) and (**b**) the binary CGH with different sizes (1 inch, 2 inches, and 3 inches) at 300 dpi and 600 dpi.

To achieve this, the computed CGHs were printed on white paper at two resolutions (300 dpi and 600 dpi), and each resolution was scaled to different sizes (1 inch, 2 inches, and 3 inches). The effect of changing the printing resolution and the size of the CGH is shown in Figure 7. The quality of the numerical reconstruction for each case was assessed quantitatively by estimating the speckle signal-to-noise ratio (SSNR). The SSNR is defined as the ratio of the standard deviation to the mean pixel intensity within a region of interest. It is often used as a measure of the speckle noise level relative to the signal strength within that region. A higher SSNR indicates a stronger signal relative to the noise. Figure 8 shows the estimated SSNR for the two printing resolutions (300 dpi and 600 dpi) with different sizes (1 inch, 2 inches, and 3 inches). The results show that the reconstruction of the grayscale CGH that had a size of 3 inches (600 dpi) gave the worst SSNR (0.3469), while the reconstruction of the binary CGH with a resolution of 600 dpi and a size of 3 inches yielded the best SSNR (0.7021).

This indicates that the recommended criteria for obtaining adequate reconstruction quality is that the binary form of CGH should be used for printing and scanning, with a size starting from 2 inches and a resolution of 600 dpi. This proves that a direct relationship exists between the printing size, resolution, and form of the CGH image and the quality of the numerical reconstruction of the CGH.

## 4. Conclusions

A new CGH-based approach for securing valuable physical and digital documents has been proposed. The method uses IFTA integrated with the XOR encryption process to generate a phase-only CGH for a document of interest. The obtained grayscale CGH was converted to a binary one to evaluate the quality of digital reconstruction of both grayscale and binary forms in terms of SSNR. The reconstruction process of the calculated CGH was performed via IFFT followed by XOR decryption. The results show that the binary CGH has a better reconstruction quality than the grayscale one. In addition, the effect of changing the printing size and the resolution of the printed CGH was investigated, showing a significant improvement in reconstruction results with increasing printed hologram size. Moreover, the security strength against cryptographic attacks was evaluated using entropy, horizontal correlation, NPCR, and UACI, revealing robust protection against brute-force attacks. In conclusion, our proposed method provides a practical and reliable solution for the security of valuable documents. This overcomes the limitations of other storage methods like QR codes. The integration of XOR encryption and decryption processes with the calculated CGH enhances data confidentiality and integrity. Furthermore, our approach is cost-effective and can be implemented using commonly available devices such as office laser printers, smartphones, and scanners, eliminating the need for specialized holographic equipment. This accessibility makes CGH-based security features more widely accepted, enabling various entities such as corporations, individuals, governments, and organizations to enhance the security of their important documents. Reducing the risks associated with counterfeiting, forgery, and unauthorized access ultimately contributes to the achievement of the SDGs.

## Figures and Tables

**Table 1:** Example of encryption and decryption of binary CGH matrix row using XOR technique with symmetric encryption and decryption key. The table shows all the steps required to encrypt the row, the encryption/decryption key used, the resulting encryption, referred to as the Cipher matrix, and the result of the decryption, referred to as the Plain text matrix. Note that these operations are performed based on the model described by Equations (6) and (7).

Matrix of binary CGH image	1	0	1	0	1	1	0	0	1	1	0	0	1	1	1	0	0	1	1
Encryption key	1	1	0	1	0	0	1	1	0	1	0	0	1	1	0	1	0	0	1
Cipher matrix	0	1	1	1	1	1	1	1	1	0	0	0	0	0	1	1	0	1	0
Decryption key	1	1	0	1	0	0	1	1	0	1	0	0	1	1	0	1	0	0	1
Plain matrix	1	0	1	0	1	1	0	0	1	1	0	0	1	1	1	0	0	1	1

**Table 2:** Statistical security tests for the original and XOR-encrypted CGHs using different metrics: entropy, horizontal correlation, number of pixels change rate (NPCR), and unified average changing intensity (UACI).

Metric	Original Image	Encrypted Image 1	Encrypted Image 2
Entropy	1.0000	1.0000	1.0000
Horizontal correlation	0.3579	−0.0012	0.0010
NPCR (number of pixels change rate) %	-	50.0338	50.0338
UACI (unified average changing intensity) %	-	50.0338	50.0338

## Data Availability

The raw data supporting the conclusions of this article will be made available by the authors upon request.
